# CCAIB: Congestion Control Based on Adaptive Integral Backstepping for Wireless Multi-Router Network

**DOI:** 10.3390/s22051818

**Published:** 2022-02-25

**Authors:** Xiaoping Deng, Lujuan Ma, Xiaoping Liu

**Affiliations:** 1 Shandong Key Laboratory of Intelligent Buildings Technology, School of Information and Electrical Engineering, Shandong Jianzhu University, Jinan 250101, China; dengxiaoping19@sdjzu.edu.cn; 2 Faculty of Engineering, Lakehead University, Thunder Bay, ON P7B 5E1, Canada; xliu2@lakeheadu.ca

**Keywords:** congestion control, wireless networks, adaptive integral backstepping

## Abstract

Wireless information collecting and processing terminals, such as cell phones, sensors and smart wearable devices, are expected to be deployed on a large scale in the future to promote the continuous advancement of the global information revolution. Since most of these terminals connect to each other using long-distance and high-speed networks by multiple routers and eventual access the internet, the application of mobile internet is gradually increasing and data traffic on the mobile internet is growing exponentially, from which arises congestion in wireless networks on multiple routers. This research solves the congestion problem for wireless networks with multiple bottleneck routers. First, the wireless network model is expanded to multi-router networks, which considers the interrelationships between connecting routers. Afterwards, a new Active Queue Management (AQM) method called Congestion Control Based on Adaptive Integral Backstepping (CCAIB) is designed to handle congestion in wireless networks. In CCAIB, an adaptive control method is used to estimate the packet loss ratios of wireless links and a controller is designed based on the estimation results through a backstepping procedure. It can be shown from the simulation results that the performance of CCAIB is better than the H*∞* algorithm in queue length stability. Besides, the window size of CCAIB is 100 times that of the H*∞* algorithm, and the proportion of packets marked as discarded when using CCAIB is about 0.1% of the H*∞* algorithm. Moreover, CCAIB has satisfactory adaptability to network parameters such as wireless link capacity, propagation delay, wireless packet loss ratios, desired queue length and router location.

## 1. Introduction

With the advent of the IoT and 5G era, more and more data traffic will flood into the Internet through wireless links, and wireless network congestion will become increasingly severe. Congestion control problems can be tackled from different perspectives, one of which is based on control methods and the other based on optimization theory. Congestion control based on control methods can be further divided into two main categories. One class is source-based, which is deployed on terminals, and the other is router-based, which is conducted by routers. There are alsovarious specific congestion control algorithms in each class. Vegas [1] is a typical source-based control algorithm, which determines the amount of bandwidth that is available for the networks via Round-Trip Time (RTT), and adjusts the window size accordingly. This is different from another source-based strategy, New Reno [2], which estimates the available bandwidth through whether acknowledgement (ACK) is received. The window size of [3] depends on the maximum window size of the last congestion and the time interval from the last congestion. Bottleneck bandwidth and round-trip time congestion control (BBR) [4] is proposed by Google, which believes that congestion occurs when the amount of packets of the networks is bigger than the product of the bottleneck link bandwidth and the delay. Ref. [5] constructs a model using artificial intelligence (AI). Ref. [6] shows that source-based control strategies have limitations such as blindness, passiveness, and delay. For the above reasons, control methods deployed on routers are advised by Internet Engineering Task Force (IETF) [7]. The Random Early Detection (RED) algorithm [8] discards new packets with a certain ratio when the queue length is over a certain value. Afterwards, a series of improved algorithms of RED are proposed [9,10,11,12,13,14,15]. However, the performance of the RED algorithms vary greatly when different parameters are selected [16]. In order to achieve more stable performance, Kathleen Nichols proposed an algorithm to achieve fairer congestion control by controlling queue delay rather than queue length [17]. For the same purpose, Rong Pan introduced a new algorithm, which can adjust the parameters adaptively [18].

After the fluid model [19] is proposed, some new router-based control algorithms [20,21,22], are applied to congestion control. Many of them are adopted in the control design of AQM mechanisms to handle the changing and uncertainty of the network parameters. Ref. [23] focuses on link capacity variance and model uncertainty. Topology uncertainty is considered in [24]. Some researchers use advanced control design methods, such as neural networks control [25], fuzzy control [26], predictive control [27,28,29] and so on. In addition to the above-mentioned algorithms based on the control methods, Srisankar [30], Xidong Deng [31] and S. Athuraliya [32] introduce optimization theory to solve the congestion problem.

Backstepping is a typical control design method for nonlinear systems [33,34,35,36,37,38], which designs a controller for nonlinear systems in two steps. Lyapunov functions are designed for each step and the two steps are connected by a virtual control law. Backstepping is commonly used in complex systems and it has been widely used in AQM for the last few years. Ref. [39] combines backstepping, H*∞*, adaptive control and prescribed performance control (PPC) to design a queue tracking controller. In [40], minimax together with integral backstepping is used, which considers user datagram protocol (UDP) flows as disturbances. Input and output saturations are both noted in [41]. Refs. [42,43,44,45] use a model with changing RTT to design controllers. Backstepping sliding mode control is used in [46]. Ref. [47] applies H*∞* control and integral backstepping to a model with both internal and external disturbances. The Transmission Control Protocol (TCP) Vegas model is used in [48]. Ref. [49] incorporates funnel control and fuzzy logic into backstepping method.

All the above research is related to a single congestion router. However, data travels through multiple routers to the receiving end in the real networks, and with the increase in data traffic on the internet, simultaneous congestion of multiple routers is common. Ref. [50] proves that congestion control algorithms designed for single bottleneck router networks are not feasible in multiple bottleneck routers networks. Multiple bottleneck routers are considered in very few studies. Refs. [51,52] use Linear Matrix Inequality (LMI) to study the stability of the networks in which congestion occurs on multiple routers. However, network congestion is a typical nonlinear problem.

Besides, all the above algorithms are proposed for wired internet, which believe all packet loss is caused by congestion. This is false in wireless networks because packet loss can also occur in the wireless links. When packet loss occurs in the wireless link, the congestion control algorithms for wired networks may assume that congestion occurs mistakenly, thus causing excessive packet loss in the queue. Ref. [53] introduces a wireless network model by modifying the fluid model, and then gives a H*∞* method after approximate linearization. After that, a few pieces of research emerged based on the new model. Fractional order proportional integral and small signal theory are used in [54]. Ref. [55] proposes a linear quadratic method to deal with the wireless AQM problem. Ref. [56] extends the backstepping control strategy to wireless TCP network to solve the AQM problem.

It is anticipated that data traffic of mobile internet will grow exponentially, which will create congestion in wireless networks on multiple routers. However, as far as the authors know, existing algorithms designed for wireless networks congestion control are based on approximate linearized models, which are distortion compared to the actual networks. Besides, to the best of our knowledge, there is little few research on wireless networks with congestion occurring on multiple routers.

The main contributions of this paper can be summarized as follows.

1. A wireless network model is introduced, in which a serial topology is considered and the interrelationships between connecting routers is formulated.
2. The adaptive control theory is applied to estimate the packet loss ratios of the uplink and downlink. Based on the estimation, a novel congestion control algorithm called CCAIB is designed using the integral backstepping procedure. Different from the previous work for wired networks, the packet loss ratios of the wireless links are taken into account.
3. Comparison is conducted between CCAIB and H*∞* in the performance of queue length stability, window size and packet loss ratio in the queue.
4. The influence of network parameters such as link capacity, propagation delay, wireless packet loss ratios, desired queue length and router location on the performance of CCAIB are analyzed.

The rest of this paper is arranged as follows. In Section 2, the topology and mathematical model of wireless networks is given. The controller design process is described in Section 3. Performance evaluation is carried out in Section 4. A conclusion is given in Section 5.

## 2. Congestion Control Model for Wireless Multi-Router Networks

**Figure 1:**
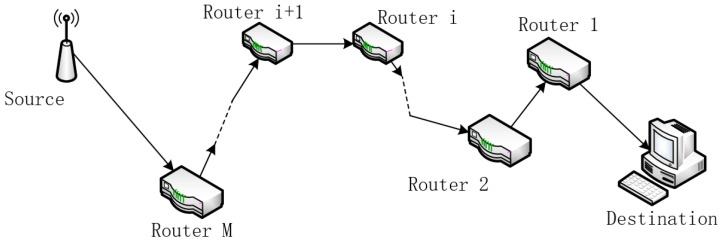
Network model. The data packets generated by sources such as sensors are relayed one by one in a serial manner through multiple wireless routers and finally delivered to the destination.

Figure 1 shows the topology, which is supposed to be series.

Different from other models, ref. [53] formulates the packet loss ratios of wireless links and this is more in line with the actual wireless networks. However, ref. [53] does not take multiple bottleneck routers into account. Thus, this paper introduces the following nonlinear TCP dynamic traffic model for wireless networks with congestion occurs on multiple routers by describing the traffic relationship between adjacent routers.

(1) $$\left\{\begin{array}{ccc} {\dot{W}}_{i}\left(t\right) & = & \frac{1}{R_{i}\left(t\right)}\left(1-p_{i}\left(t\right)\right)-\left(1-p_{{}_{i},dl}\left(t\right)\right)\frac{W_{i}\left(t\right)}{2}\frac{W_{i}\left(t\right)}{R_{i}\left(t\right)}p_{i}\left(t\right)\\ \; & \; & -p_{i,dl}\left(t\right)\left(W_{i}\left(t\right)-1\right)\frac{W_{i}\left(t\right)}{R_{i}\left(t\right)}p_{i}\left(t\right)\\ {\dot{q}}_{i}\left(t\right) & = & \frac{W_{i}\left(t\right)}{R_{i}\left(t\right)}\left(1-p_{i,ul}\left(t\right)\right)-C_{i}\left(t\right)\\ R_{i}\left(t\right) & = & T_{p}+\frac{q_{i}\left(t\right)}{C_{i}\left(t\right)} \end{array}\right.$$

where

(2) $$C_{i}\left(t\right)=\left\{\begin{array}{c} C_{1}\;\;\;\;\;\left(i=1\right)\\ \frac{W_{i-1}\left(t\right)}{R_{i-1}\left(t\right)}\;\;\;\left(i⩾2\right) \end{array}\right.$$

is the link capacity after router *i* (i=1,2,3,…,M) and *M* is the number of bottleneck routers. Meanings and ranges of symbols in (1) are listed in Table 1.

Assume the receiving capacity of the destination terminal is fixed, thus $C_{1}$ can be considered constant, as shown in (2). This assumption fails when i⩾2 because $C_{i}$ is related to $W_{i-1}\left(t\right)$ and $R_{i-1}\left(t\right)$, as shown in (2). In the following derivations, the time functions are replaced by variables.

The first equation of (1) formulates the derivative of the window size for each router in time. The first term $\frac{1}{R_{i}\left(t\right)}\left(1-p_{i}\left(t\right)\right)$ shows that when an ACK is received within an RTT, the window size is increased by 1 and this is the congestion avoidance mechanism in TCP. The second term $\left(1-p_{dl,i}\left(t\right)\right)\frac{W_{i}\left(t\right)}{2}\frac{W_{i}\left(t\right)}{R_{i}\left(t\right)}p_{i}\left(t\right)$ shows that when a Negative Acknowledgement (NACK) is received within an RTT, the window size is reduced to half of its original size and this is the fast recovery procedure in TCP. The third term $p_{dl,i}\left(t\right)\left(W_{i}\left(t\right)-1\right)\frac{W_{i}\left(t\right)}{R_{i}\left(t\right)}p_{i}\left(t\right)$ represents neither ACK nor NACK has received within an RTT. In this case, the source uses the latest packet loss ratio and subtracts 1 from the window size.

The second equation of (1) describes the derivative of queue length in time, which is equal to the rate of packet increase minus the rate of packet decrease in the queue. The meaning of $1-p_{ul,i}\left(t\right)$ is the probability of receiving ACK.

The third equation of (1) gives the calculation method of RTT, which is composed of propagation time and queue time.

Since active discarding can select packets that are relatively less important to discard, QAM actively drops a certain percentage of packets to keep the queue at a certain length to avoid packets being passively dropped. Queue packet loss ratio is a ratio where the numerator is the number of packets that need to be actively dropped from the queue, and the denominator is the total number of packets in the queue. The design idea of the controller in this paper is to tune the value of queue packet loss ratio that can keep the queue at a specific length, that is, the error between the expected length and the actual length is 0.

This paper is to propose an algorithm satisfying: (1) the packet loss ratios of uplink $p_{ul,i}\left(t\right)$ and downlink $p_{dl,i}\left(t\right)$ are estimated; (2) the queue length $q_{i}\left(t\right)$ is stable at the desired queue length $q_{i,r}$ well; (3) the window size $W_{i}\left(t\right)$ is as large as possible so as to maximize throughput; (4) the queue packet loss ratio being marked as dropped is as small as possible.

In order to facilitate the derivations, the following redefinitions have been carried out.

Denote $x_{i,1}=q_{i}-q_{i,r}$, $x_{i,2}=W_{i}$, $u_{i}=p_{i}$, where $q_{i,r}$ is the desired queue length for router *i*. The integral term is defined as $x_{i,0}=\int_{0}^{t}\left(q_{i}\left(\tau \right)-q_{i,r}\left(\tau \right)\right)d\tau$. $\theta_{i,1}=p_{ul,i}$ and $\theta_{i,2}=p_{dl,i}$. Then, (1) can be rewritten as:

(3) $$\left\{\begin{array}{cc} {\dot{x}}_{i,0}= & x_{i,1}\\ {\dot{x}}_{i,1}= & f_{i,1}\left(x_{i,1}\right)+\left(g_{i,10}\left(x_{i,1}\right)+g_{i,11}\left(x_{i,1}\right)\theta_{i,1}\right)x_{i,2}\\ {\dot{x}}_{i,2}= & f_{i,2}\left(x_{i,1}\right)+\left(g_{i,20}\left(x_{i,1},x_{i,2}\right)+g_{i,21}\left(x_{i,1},x_{i,2}\right)\theta_{i,2}\right)u_{i} \end{array}\right.$$

where $f_{i,1}=-C_{i}-{\dot{q}}_{i,r}$, $f_{i,2}=\frac{1}{R_{i}}$, $g_{i,10}=\frac{1}{R_{i}}$, $g_{i,11}=-\frac{1}{R_{i}}$, $g_{i,20}=-\frac{1}{R_{i}}-\frac{W_{i}}{2}\frac{W_{i}}{R_{i}}$, and $g_{i,21}=\frac{W_{i}}{R_{i}}\left(1-\frac{W_{i}}{2}\right)$.

Based on (3), the congestion controller based on adaptive integral backstepping is designed in the following section.

## 3. CCAIB Algorithm

Backstepping is a control method based on Lyapunov’s second method. That is, if a Lyapunov function is designed to be positive definite and its differential is negative definite, then the quadratic terms of the Lyapunov function can be proved to converge to zero. In the first step of CCAIB, a virtual control law is used to construct the quadratic term of Lyapunov function. In the second step of CCAIB, the queue length convergence error is the quadratic term of Lyapunov function. By these two steps, the queue length is stable at a certain value.

Step 1:

Choose a Lyapunov function as

(4) $$V_{i,1}=\frac{1}{2}K_{i,0}x_{i,0}^{2}+\frac{1}{2}x_{i,1}^{2}+\frac{1}{2}\Gamma_{i,1}{\left(\theta_{i,1}-{\hat{\theta }}_{i,1}\right)}^{2}$$

with $K_{i,0}$ and $\Gamma_{i,1}$ being positive design parameters and ${\hat{\theta }}_{i,1}$ being the estimation of $\theta_{i,1}$. The purpose of introducing the Lyapunov function is that if we can prove that the Lyapunov function is positive definite, and its derivative is negative definite, then the value of the Lyapunov function will converge to 0. If each of its terms is nonnegative, then each of its terms will converge to 0. Specifically, for the last term of the Lyapunov function, this means the error between the actual and the estimated packet loss ratios of wireless links will converge to zero, and the target of estimating the packet loss ratios of wireless links will be satisfied. It is easily seen that $V_{i,1}$ is positive definite. Then, it needs to be proved that ${\dot{V}}_{i,1}$ is negative definite.

Differentiating $V_{i,1}$ gives

(5) $$\begin{array}{ccc} {\dot{V}}_{i,1} & = & K_{i,0}x_{i,0}x_{i,1}+x_{i,1}\left[f_{i,1}+\left(g_{i,10}+g_{i,11}\theta_{i,1}\right)x_{i,2}\right]+\Gamma_{i,1}\left(\theta_{i,1}-{\hat{\theta }}_{i,1}\right)\left(-{\overset{\cdot }{\hat{\theta }}}_{i,1}\right)\\ & = & x_{i,1}[K_{i,0}x_{i,0}+f_{i,1}+\left(g_{i,10}+g_{i,11}\theta_{i,1}\right)\left(x_{i,2}-\alpha_{i,1}\right)\\ & & +\left(g_{i,10}+g_{i,11}{\hat{\theta }}_{i,1}+g_{i,11}\left(\theta_{i,1}-{\hat{\theta }}_{i,1}\right)\right)\alpha_{i,1}]+\Gamma_{i,1}\left(\theta_{i,1}-{\hat{\theta }}_{i,1}\right)\left(-{\overset{\cdot }{\hat{\theta }}}_{i,1}\right)\\ & = & x_{i,1}\left[K_{i,0}x_{i,0}+f_{i,1}+\left(g_{i,10}+g_{i,11}{\hat{\theta }}_{i,1}+g_{i,11}\left(\theta_{i,1}-{\hat{\theta }}_{i,1}\right)\right)\alpha_{i,1}\right]\\ & & +x_{i,1}\left(g_{i,10}+g_{i,11}\theta_{i,1}\right)\left(x_{i,2}-\alpha_{i,1}\right)+\Gamma_{i,1}\left(\theta_{i,1}-{\hat{\theta }}_{i,1}\right)\left(-{\overset{\cdot }{\hat{\theta }}}_{i,1}\right)\\ & = & x_{i,1}\left[K_{i,0}x_{i,0}+f_{i,1}+\left(g_{i,10}+g_{i,11}{\hat{\theta }}_{i,1}\right)\alpha_{i,1}\right]\\ & & +x_{i,1}\left(g_{i,10}+g_{i,11}\theta_{i,1}\right)\left(x_{i,2}-\alpha_{i,1}\right)\\ & & +\left(\theta_{i,1}-{\hat{\theta }}_{i,1}\right)\left(x_{i,1}\alpha_{i,1}g_{i,11}-\Gamma_{i,1}{\overset{\cdot }{\hat{\theta }}}_{i,1}\right) \end{array}$$

To ensure that ${\dot{V}}_{i,1}$ satisfies negative definiteness, we set

(6) $$\begin{array}{ccc} K_{i,0}x_{i,0}+f_{i,1}+\left(g_{i,10}+g_{i,11}{\hat{\theta }}_{i,1}\right)\alpha_{i,1} & = & -K_{i,1}x_{i,1} \end{array}$$

(7) $$\begin{array}{ccc} x_{i,1}\alpha_{i,1}g_{i,11}-\Gamma_{i,1}{\overset{\cdot }{\hat{\theta }}}_{i,1} & = & 0 \end{array}$$

where $K_{i,1}⩾0$$\left(i=1\cdots M\right)$ are design parameters.

A simple calculation yields:

(8) $$\begin{array}{ccc} \alpha_{i,1} & = & \frac{-K_{i,0}x_{i,0}-K_{i,1}x_{i,1}-f_{i,1}}{g_{i,10}+g_{i,11}{\hat{\theta }}_{i,1}} \end{array}$$

(9) $$\begin{array}{ccc} {\overset{\cdot }{\hat{\theta }}}_{i,1} & = & \Gamma_{i,1}^{-1}x_{i,1}\alpha_{i,1}g_{i,11} \end{array}$$

Substituting (8) and (9) into (5) leads to

(10) $${\dot{V}}_{i,1}=-K_{i,1}x_{i,1}^{2}+x_{i,1}\left(g_{i,10}+g_{i,11}\theta_{i,1}\right)\left(x_{i,2}-\alpha_{i,1}\right)$$

Step 2:

Constructing a Lyapunov function

(11) $$\begin{array}{ccc} V_{i,2} & = & V_{i,1}+\frac{1}{2}{\left(x_{i,2}-\alpha_{i,1}\right)}^{2}+\frac{1}{2}\Gamma_{i,2}{\left(\theta_{i,2}-{\hat{\theta }}_{i,2}\right)}^{2}+\frac{1}{2}{\overset{ˇ}{\Gamma }}_{i,1}{\left(\theta_{i,1}-{\overset{ˇ}{\theta }}_{i,1}\right)}^{2} \end{array}$$

where $\Gamma_{i,2}>0$ and ${\overset{ˇ}{\Gamma }}_{i,1}>0$ are both constants. $\theta_{i,1}$ and $\theta_{i,2}$ are estimated by ${\overset{ˇ}{\theta }}_{i,1}$ and ${\hat{\theta }}_{i,2}$ respectively.

Differentiating $V_{i,2}$ results in

(12) $$\begin{array}{ccc} {\dot{V}}_{i,2} & = & -K_{i,1}x_{i,1}^{2}+x_{i,1}\left(g_{i,10}+g_{i,11}\theta_{i,1}\right)s_{i,2}\\ & & +s_{i,2}\left({\dot{x}}_{i,2}-{\dot{\alpha }}_{i,1}\right)+\left(\theta_{i,2}-{\hat{\theta }}_{i,2}\right)\left(-\Gamma_{i,2}{\overset{\cdot }{\hat{\theta }}}_{i,2}\right)\\ & & +\left(\theta_{i,1}-{\overset{ˇ}{\theta }}_{i,1}\right)\left(-{\overset{ˇ}{\Gamma }}_{i,1}{\overset{\cdot }{\overset{ˇ}{\theta }}}_{i,1}\right)\\ & = & -K_{i,1}x_{i,1}^{2}+x_{i,1}\left(g_{i,10}+g_{i,11}\theta_{i,1}\right)s_{i,2}\\ & & +s_{i,2}\left(z_{i,2}+\left(g_{i,20}+g_{i,21}\theta_{i,2}\right)u_{i}\right)\\ & & -s_{i,2}\left(\frac{\partial \alpha_{i,1}}{\partial x_{i,1}}\left(f_{i,1}+\left(g_{i,10}+g_{i,11}\theta_{i,1}\right)x_{i,2}\right)\right)\\ & & +\left(\theta_{i,2}-{\hat{\theta }}_{i,2}\right)\left(-\Gamma_{i,2}{\overset{\cdot }{\hat{\theta }}}_{i,2}\right)+\left(\theta_{i,1}-{\overset{ˇ}{\theta }}_{i,1}\right)\left(-{\overset{ˇ}{\Gamma }}_{i,1}{\overset{\cdot }{\overset{ˇ}{\theta }}}_{i,1}\right)\\ & = & -K_{i,1}x_{i,1}^{2}+s_{i,2}x_{i,1}g_{i,10}\\ & & +s_{i,2}\left(x_{i,1}g_{i,11}-n_{i,2}\right)\left(\theta_{i,1}-{\overset{ˇ}{\theta }}_{i,1}\right)+s_{i,2}\left(x_{i,1}g_{i,11}-n_{i,2}\right){\overset{ˇ}{\theta }}_{i,1}\\ & & +s_{i,2}\left[z_{i,2}+\left(g_{i,20}+g_{i,21}\left(\theta_{i,2}-{\hat{\theta }}_{i,2}\right)\right)u_{i}\right]+s_{i,2}\left({\hat{\theta }}_{i,2}g_{i,21}u_{i}-m_{i,2}\right)\\ & & +\left(\theta_{i,2}-{\hat{\theta }}_{i,2}\right)\left(-\Gamma_{i,2}{\overset{\cdot }{\hat{\theta }}}_{i,2}\right)+\left(\theta_{i,1}-{\overset{ˇ}{\theta }}_{i,1}\right)\left(-{\overset{ˇ}{\Gamma }}_{i,1}{\overset{\cdot }{\overset{ˇ}{\theta }}}_{i,1}\right)\\ & = & -K_{i,1}x_{i,1}^{2}+s_{i,2}x_{i,1}g_{i,10}+s_{i,2}\left(x_{i,1}g_{i,11}-n_{i,2}\right){\overset{ˇ}{\theta }}_{i,1}\\ & & +s_{i,2}\left(z_{i,2}+g_{i,20}u_{i}+{\hat{\theta }}_{i,2}g_{i,21}u_{i}-m_{i,2}\right)\\ & & +\left(\theta_{i,1}-{\overset{ˇ}{\theta }}_{i,1}\right)\left[s_{i,2}\left(x_{i,1}g_{i,11}-n_{i,2}\right)-{\overset{ˇ}{\Gamma }}_{i,1}{\overset{\cdot }{\overset{ˇ}{\theta }}}_{i,1}\right]\\ & & +\left(\theta_{i,2}-{\hat{\theta }}_{i,2}\right)\left(s_{i,2}g_{i,21}u_{i}-\Gamma_{i,2}{\overset{\cdot }{\hat{\theta }}}_{i,2}\right) \end{array}$$

where $z_{i,2}=f_{i,2}-\frac{\partial \alpha_{i,1}}{\partial x_{i,0}}x_{i,1}-\frac{\partial \alpha_{i,1}}{\partial {\hat{\theta }}_{i,1}}{\overset{\cdot }{\hat{\theta }}}_{i,1}$, $s_{i,2}=x_{i,2}-\alpha_{i,1}$, $m_{i,2}=\frac{\partial \alpha_{i,1}}{\partial x_{i,1}}\left(f_{i,1}+g_{i,10}x_{i,2}\right)$ and $n_{i,2}=\frac{\partial \alpha_{i,1}}{\partial x_{i,1}}x_{i,2}g_{i,11}$.

To ensure that ${\dot{V}}_{i,2}$ satisfies negative definiteness, we set

(13) $$\begin{array}{ccc} s_{i,2}\left(x_{i,1}g_{i,11}-n_{i,2}\right)-{\overset{ˇ}{\Gamma }}_{i,1}{\overset{\cdot }{\overset{ˇ}{\theta }}}_{i,1} & = & 0 \end{array}$$

(14) $$\begin{array}{ccc} s_{i,2}g_{i,21}u_{i}-\Gamma_{i,2}{\overset{\cdot }{\hat{\theta }}}_{i,2} & = & 0 \end{array}$$

(15) $$\begin{array}{c} x_{i,1}g_{i,10}+\left(x_{i,1}g_{i,11}-n_{i,2}\right){\overset{ˇ}{\theta }}_{i,1}+z_{i,2}\\ =m_{i,2}-K_{i,2}s_{i,2}-\left(g_{i,20}+{\hat{\theta }}_{i,2}g_{i,21}\right)u_{i} \end{array}$$

It can be obtained from the above equation that:

(16) $$\begin{array}{ccc} {\overset{\cdot }{\overset{ˇ}{\theta }}}_{i,1} & = & {\overset{ˇ}{\Gamma }}_{i,1}^{-1}s_{i,2}\left(x_{i,1}g_{i,11}-n_{i,2}\right) \end{array}$$

(17) $$\begin{array}{ccc} {\overset{\cdot }{\hat{\theta }}}_{i,2} & = & \Gamma_{i,2}^{-1}s_{i,2}g_{i,21}u_{i} \end{array}$$

(18) $$u_{i}=\frac{-x_{i,1}g_{i,10}-\left(x_{i,1}g_{11}-n_{i,2}\right){\overset{ˇ}{\theta }}_{i,1}+m_{i,2}-z_{i2}-K_{i,2}s_{i,2}}{g_{i,20}+{\hat{\theta }}_{i,2}g_{i,21}}$$

Substituting (16)–(18) into (12) gives

(19) $${\dot{V}}_{i,2}=-K_{i,1}x_{i,1}^{2}-K_{i,2}^{2}s_{i,2}^{2}$$

Hence, $x_{i,1}$ and $s_{i,2}$ are both proved to converge to zero.

By now, with the controller designed above, the queue length can track the desired queue length well.

## 4. Performance Evaluation

To show the effectiveness and superiority of CCAIB, simulations are conducted. First of all, comparison between CCAIB and H*∞* approaches proposed in [53] is conducted. H*∞* is an effective tool to deal with external disturbance and uncertainty in time delay. When adopting H*∞* algorithm in congestion control problems, the queue length of the router will be maintained at targeted level, which is implied by the asymptotical stability of the system, with minimum sensitivity to the fluctuation of the available link bandwidth [53]. A detailed description of this algorithm can be found in reference [53] and the references therein.

Algorithm proposed in [53] is chosen as the comparison algorithm because it is also designed for wireless networks. Many of the classic congestion control algorithms have not been used for comparison because it makes little sense to compare algorithms designed for wired networks with algorithms designed for wireless networks. Besides, although there have been some literatures dealing with congestion control in wireless networks scenarios, in almost these studies, except [53] to the best of our knowledge, the wireless networks are modeled using linear model, which cannot reflect actual behavior and dynamics of realistic wireless networks with high fidelity. Besides, ref. [53] considers the wireless access networks consisting of only one bottleneck router and the model is constructed accordingly. In this work, the wireless network model is expanded to multi-router networks which consider the inter-relationships between connecting routers. Thus, the comparison is implemented only on a single router. Afterwards, CCAIB is simulated with multiple bottleneck routers and impact of network parameters on CCAIB is evaluated.

### 4.1. Network Topology and Parameters

The network topology is shown in Figure 1. For simplicity, multiple devices and sessions are represented by one source in the figure.

In the comparison between CCAIB and H*∞*, four cases are investigated and the network parameters are shown in Table 2. The desired queue length is 100 packets for all four cases. We set the link capacity to 1000 packets/s and 3000 packets/s to represent low-speed and high-speed transmission scenarios, respectively. In the real wireless networks, for example, mobile network and Wi-Fi, the wireless packet loss ratios are usually lower than 5%. Thus, 0.05 and 0.005 are chosen to be the packet loss ratios for high quality link and low quality link, respectively [57]. It is worth noting that the wireless packet loss ratios are unknown for the system and to be estimated in CCAIB. In the evaluation of impact of network parameters, one network parameter varies and the other parameters are set according to Table 3. The controller parameters of CCAIB are set as Table 4. Parameters of H*∞* are set the same as [53]. The three key performance indicators (KPIs) used to compare CCAIB and H*∞* algorithm are the convergence performance of the queue, the window size and packet loss ratio in the queue.

### 4.2. Results and Analysis

In this subsection, simulation results are demonstrated and discussed. For graphs encompassing a wide range, logarithmic Y-axis scales are used for readability. In some graphs, a magnified graph is superimposed to show the detail. Except for the simulation for different routers, other groups only show the results of router 1 since all the routers have same trends.

#### 4.2.1. Comparison between CCAIB and H*∞* Algorithm

**Figure 2:**
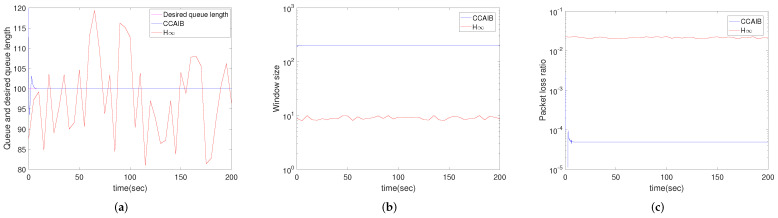
Simulation results of single router using CCAIB and H*∞* algorithm for case 1. Columns from left to right show the performance of CCAIB and H*∞* in (**a**) queue length stability, (**b**) window size and (**c**) packet loss ratio in the queue.

Figure 2 demonstrates the performance of CCAIB and H*∞* in queue length stability, window size and packet loss ratio in the queue for case 1. Results for the other three cases are not shown to save space because they have the same trends as case 1. It is observed that the queue length of CCAIB converges in 10 s but that of H*∞* is misconvergence throughout the time. CCAIB’s queue packet loss ratio is about 0.1% of H*∞* algorithm. This is because for CCAIB, packet loss ratios of the wireless links are estimated and are taken into account when determining the packet loss ratio in queue, which improves the networks performance obviously. The window size of CCAIB is about 100 times that of the H*∞* algorithm. This is easy to understand because the queue packet loss ratio of CCAIB is much smaller than the H*∞* algorithm. Besides, the H*∞* algorithm causes larger and more jitters in queue packet loss ratio and window size.

#### 4.2.2. Simulation Results for Different Link Capacity

**Figure 3:**
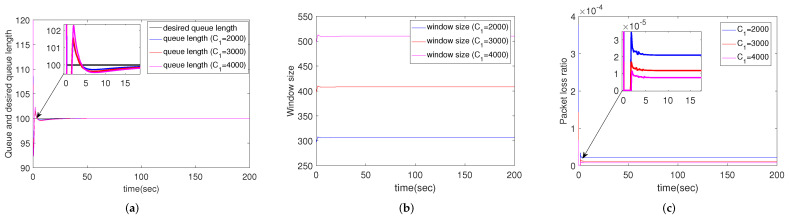
Simulation results of multiple routers using CCAIB for different link capacities. Columns from left to right show the performance of CCAIB in (**a**) queue length stability, (**b**) window size and (**c**) packet loss ratio in the queue.

Figure 3 depicts the simulation results of scenarios when the bandwidth $C_{1}=2000$, 3000, 4000 packets/s respectively. To show the details, the enlarged images are superimposed in Figure 3a,c. The following subsections use the same method to show the details. Figure 3a shows that the queue length can converge within 15 s regardless of the link capacity. As shown in Figure 3c, although the bandwidth is different, the queue packet loss ratios are under $6\times 10^{-4}$ (under $3\times 10^{-5}$ in stable state). Figure 3b shows that the window sizes become stable with different bandwidths. In addition, a larger bandwidth corresponds to lower packet loss ratio and larger window size. That is to say, we can improve the effectiveness (lager window size) and reliability (lower packet loss ratio) of the networks by increasing the bandwidth.

#### 4.2.3. Simulation Results for Different Propagation Delays

**Figure 4:**
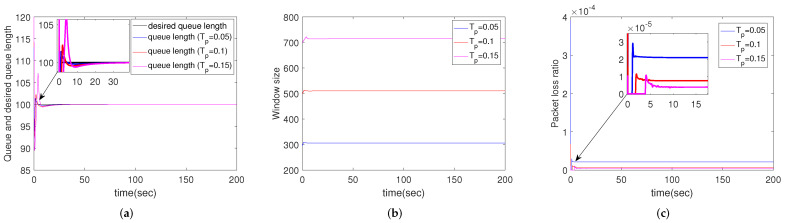
Simulation results of multiple routers using CCAIB for different propagation delays. Columns from left to right show the performance of CCAIB in (**a**) queue length stability, (**b**) window size and (**c**) packet loss ratio in the queue.

In this group, $T_{p}=0.05$, 0.1, 0.15 s, respectively. Figure 4 shows the performance of different propagation delays. Although all the queue lengths converge in 15 s with different propagation delays, a bigger propagation delay leads to more serious overshoot, as shown in Figure 4a. It is obvious in Figure 4b,c that a bigger propagation delay also produces larger window size and lower packet loss ratio. That is because when the packets arrive to the router slowly, the congestion can be alleviated. It should be noted that larger propagation delay decreases the effectiveness of the communication. So, there must be a tradeoff when the distance between routers is determined.

#### 4.2.4. Simulation Results for Different Packet Loss Ratios of Uplink

**Figure 5:**
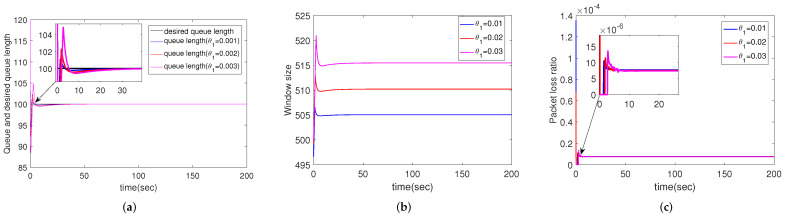
Simulation results of multiple routers using CCAIB for different uplink packet loss ratios. Columns from left to right show the performance of CCAIB in (**a**) queue length stability, (**b**) window size and (**c**) packet loss ratio in the queue.

In this scenario, all the parameters are set as default except for the packet loss ratio of uplink $\theta_{1}=0.01$, 0.02, 0.03, respectively. Figure 5 shows the performance of different packet loss ratios of uplink, ie., $\theta_{1}$. Figure 5c reveals that $\theta_{1}$ has little effect on the queue packet loss ratio. However, window size increases as $\theta_{1}$ increases, as shown in Figure 5b. This is reasonable because when the queue length is fixed, more packets need to be sent from the source to make up for the packet lost in the uplink. In addition, bigger $\theta_{1}$ leads to more serious overshoot of the queue, as shown in Figure 5a.

#### 4.2.5. Simulation Results for Different Packet Loss Ratios of Downlink

**Figure 6:**
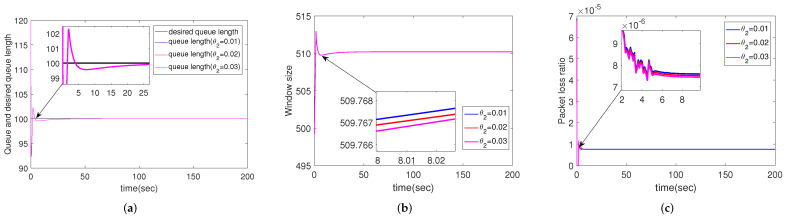
Simulation results of multiple routers using CCAIB for different downlink packet loss ratios. Columns from left to right show the performance of CCAIB in (**a**) queue length stability, (**b**) window size and (**c**) packet loss ratio in the queue.

Different from the previous subsection, the packet loss ratio of downlink changes in this subsection, while other parameters remain default. i.e., $\theta_{2}=0.01$, 0.02, 0.03, respectively. The simulation results are shown in Figure 6. Unlike $\theta_{1}$, $\theta_{2}$ has almost no effect on packet loss ratio, tracking of queue length or window size.

#### 4.2.6. Simulation Results for Different Desired Queue Length

**Figure 7:**
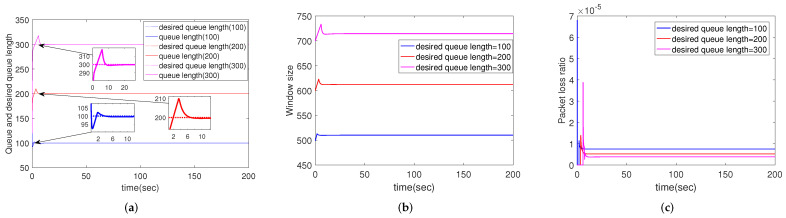
Simulation results of multiple routers using CCAIB for different desired queue lengths. Columns from left to right show the performance of CCAIB in (**a**) queue length stability, (**b**) window size and (**c**) packet loss ratio in the queue.

In this subsection, we change the desired queue length while keeping the other parameters constant, i.e., $q_{r}=100$, 200, 300 packets, respectively. From Figure 7, it is obvious that CCAIB has good convergence performance whether the desired queue length is 100, 200 or 300 packets. In addition, larger desired queue length allows for larger window size. Therefore, increasing the buffer size of the router is an effective way to increase the window size.

#### 4.2.7. Simulation Results for Different Routers

**Figure 8:**
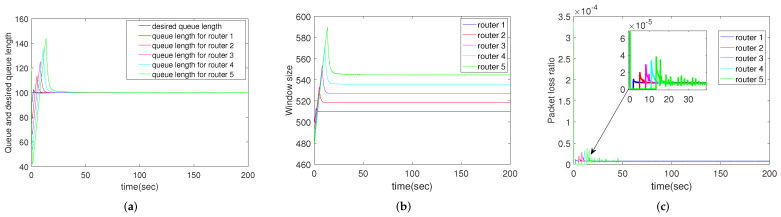
Simulation results of multiple routers using CCAIB for different routers. Columns from left to right show the performance of CCAIB in (**a**) queue length stability, (**b**) window size and (**c**) packet loss ratio in the queue.

In the above subsections, we only show the performance of router 1. In this subsection, comparison is taken between the five routers. Parameters are shown in Table 3. From Figure 8, it can be seen that router 1 has the best performance. This makes sense because the bandwidth of the router next to the receiver is determined by the receiver’s capacity, which is a constant. Other routers’ bandwidths are determined by the next-hop window size, which is constantly changing. Therefore, when routing protocols are designed, it should be with as few hops as possible.

The main conclusion obtained from the above simulation results is that the CCAIB has a superior performance than the H*∞* algorithm congestion control algorithm in queue length, convergence time, queue packet loss ratio and window size. This is basically due to the packet loss ratios of wireless links having been estimated and taken into account when determining the packet loss ratio in queue, which improves the networks performance obviously.

Through the simulation results, some technical suggestions can be provided for the deployment of the wireless networks with multiple bottleneck routers. For example, when routing protocols are designed, the number of hops between the sender and the receiver should be reduced if possible. Since a larger propagation delay decreases the effectiveness of the communication, the distance between routers should be carefully considered when the network is deployed. In addition, increasing the bandwidth and buffer size can effectively relieve the network congestion. In particular, since the quality of the uplink has a more significant impact on the performance of the entire network than the downlink, when the CCAIB is deployed, priority should be given to ensuring the quality of the uplink.

## 5. Conclusions

In this paper, we have developed a new algorithm, named CCAIB, under the framework of AQM to handle the congestion problem for wireless networks. CCAIB takes multiple bottleneck routers into account. The wireless packet loss ratios are estimated by adaptive method and the AQM problem is solved by backstepping procedure. Compared to another congestion control method for wireless networks called H*∞* algorithm, CCAIB is better in queue length convergence time, queue packet loss ratio and window size. Besides, the effectiveness and feasibility of CCAIB in different network environments are validated.

The relationship of adjacent routers is considered only for simple series topology in this research. Since different topologies have different relationships between adjacent routers, designing the control strategy for more realistic and complex network topologies, such as mesh networks, is still an open problem. Besides, this work only considers the congestion after the router, the case of congestion before router can be explored in the future work.

## Figures and Tables

**Table 1:** Meanings and ranges of symbols.

Symbols	Meanings	Ranges
$W_{i}\left(t\right)$	Window size	$\left[W_{min},W_{max}\right]$
$q_{i}\left(t\right)$	Queue length	$\left[q_{min},q_{max}\right]$
$R_{i}\left(t\right)$	RTT	$R_{+}$
$p_{i}\left(t\right)$	Queue packet loss ratio	$\left[0,1\right]$
$C_{i}\left(t\right)$	Link capacity	$R_{+}$
$T_{p}$	Propagation time	$R_{+}$
*M*	Number of congestion routers	N
$p_{dl,i}\left(t\right)$	Packet loss ratio of downlink before router *i*	$\left[0,1\right]$
$p_{ul,i}\left(t\right)$	Packet loss ratio of uplink before router *i*	$\left[0,1\right]$

**Table 2:** Network parameters for different cases.

Case	Link Capacity	Delay	Packet Loss Ratio
1	1000 packets/s	100 ms	0.005
2	3000 packets/s	100 ms	0.005
3	1000 packets/s	100 ms	0.05
4	3000 packets/s	100 ms	0.05

**Table 3:** Default network parameters.

Router location	Router 1
Link capacity	4000 packets/s
Propagation delay	100 ms
Wireless packet loss ratio	0.02
Desired queue length	100 packets

**Table 4:** Controller parameters of CCAIB.

Parameters	Values	Remark
$K_{i,0}$	10	i=1,2,3,4,5
$K_{i,1}$	10	i=1,2,3,4,5
$K_{i,2}$	10	i=1,2,3,4,5
${\overset{ˇ}{\Gamma }}_{i,0}$	$10^{7}$	i=1,2,3,4,5
${\overset{ˇ}{\Gamma }}_{i,1}$	$10^{7}$	i=1,2,3,4,5
${\overset{ˇ}{\Gamma }}_{i,2}$	$10^{7}$	i=1,2,3,4,5
